# First description of *Leishmania* (*Viannia*) infection in *Evandromyia saulensis*, *Pressatia* sp. and *Trichophoromyia auraensis* (Psychodidae: Phlebotominae) in a transmission area of cutaneous leishmaniasis in Acre state, Amazon Basin, Brazil

**DOI:** 10.1590/0074-02760160283

**Published:** 2017-01

**Authors:** Thais de Araujo-Pereira, Daniela de Pita-Pereira, Mariana Côrtes Boité, Myllena Melo, Taiana Amancio da Costa-Rego, Andressa Alencastre Fuzari, Reginaldo Peçanha Brazil, Constança Britto

**Affiliations:** 1 Fundação Oswaldo Cruz-Fiocruz, Instituto Oswaldo Cruz, Laboratório de Biologia Molecular e Doenças Endêmicas, Rio de Janeiro, RJ, Brasil; 2 Fundação Oswaldo Cruz-Fiocruz, Instituto Oswaldo Cruz, Laboratório de Pesquisa em Leishmaniose, Rio de Janeiro, RJ, Brasil; 3 Fundação Oswaldo Cruz-Fiocruz, Instituto Oswaldo Cruz, Laboratório de Doenças Parasitárias, Rio de Janeiro, RJ, Brasil

**Keywords:** sandfly, Leishmania Viannia, molecular diagnosis, Acre

## Abstract

Studies on the sandfly fauna to evaluate natural infection indexes are still limited in the Brazilian Amazon, a region with an increasing incidence of cutaneous leishmaniasis. Here, by using a multiplex polymerase chain reaction directed to *Leishmania* kDNA and hybridisation, we were able to identify *L. (Viannia)* subgenus in 12 out of 173 sandflies captured in the municipality of Rio Branco, Acre state, revealing a positivity of 6.94%. By sequencing the *Leishmania* 234 bp*-hsp70* amplified products from positive samples, infection by *L. (V.) braziliensis* was confirmed in five sandflies: one *Evandromyia saulensis*, three *Trichophoromyia auraensis* and one *Pressatia* sp. The finding of *L. (Viannia)* DNA in two *Ev. saulensis* corresponds to the first record of possible infection associated with this sandfly. Moreover, our study reveals for the first time in Brazil, *Th. auraensis* and *Pressatia* sp. infected by *L. (Viannia)* parasites.

American cutaneous leishmaniasis (ACL), characterised by single or multiple skin lesions, has been recorded in all Brazilian states and is undergoing territorial expansion, revealing changes in its epidemiological profile (MS/SVS 2010). In Brazil, the disease is caused by seven *Leishmania* species with a significant diversity of parasites found in the Amazon Basin (Ready 2013). Except in primary forests in North Brazil and the Amazon Region, *Leishmania* (*Viannia*) *braziliensis* (Vianna 1911) is the primary widespread etiologic agent of ACL in the country (Rangel & Lainson 2009). In an epidemiological context, Amazon has been identified as a circuit involving rural and occupational variables particularly associated with the destruction of forests. As a result, this region is undergoing rapid changes in environmental conditions that directly impact the population of sandfly vectors and therefore disease transmission. Knowledge of the geographical distribution of phlebotomine sandflies responsible for ACL transmission is still scarce for some regions, such as in Acre state, where the disease is endemic (Martins & Silva 1964, Arias et al. 1984, Silva-Nunes et al. 2008). Approximately 3,538 cases were reported between 2010 and 2013 in Acre, being 918 notifications registered only in the Rio Branco municipality and without records of visceral manifestation of the disease in the state (SINAN 2015). Regarding the sandfly fauna, 73 species were identified in the municipalities of Bujari, Xapuri, Rio Branco and Assis Brasil (Azevedo et al. 2008, Teles et al. 2013, Araujo-Pereira et al. 2014). Of these, ten species were recognised as *Leishmania* vectors, suggesting the existence of three transmission cycles in Acre: *L*. (*V*.) *braziliensis* by *Nyssomyia whitmani* (Antunes & Coutinho 1939), *L*. (*V*.) *lainsoni* (Silveira, Shaw, Braga & Ishikawa 1987) by *Trichophoromyia ubiquitalis* (Mangabeira 1942) and the transmission of *L*. (*V*.) *guyanensis* (Floch 1954) by *Nyssomyia umbratilis* (Ward & Fraiha 1977) (Azevedo et al. 2008). Vector surveillance investigations are often focused on studying the distribution of potential vectors and the prevalence of *Leishmania* infection in field-captured sandflies. These studies are critical to better understand the dynamics of disease transmission and to identify new parasite-vector associations.

Here we evaluate the rates of infection by *Leishmania* spp. in non-blood-fed female sandflies captured in Rio Branco municipality, Acre state, from April 2011 to April 2012, using five HP light traps (Pugedo et al. 2005) per night during fifteen nights (Araujo-Pereira et al. 2014). Collection points were distributed in forested areas impacted by the presence of man, around residences or in the Municipal Park and inside chicken enclosures in the peridomicile. Three areas with two collecting points each were considered strategic for regular captures in Rio Branco, representing sites with the occurrence of human ACL cases in the neighboring population. The study area and sandflies capture were previously described (Araujo-Pereira et al. 2014) (Figure). Four hundred and fifty six sandflies were collected (256 females and 200 males), individually mounted on glass slides and taxonomically identified into 23 species following the classification proposed by Galati (2003) with the generic abbreviations of Marcondes (2007). Taking into account the presence of blood in the gut contents of 83 insect females, a sampling consisting of 173 non-blood-fed specimens were individually analysed for the presence of *Leishmania* DNA. Genetic material was extracted from each sandfly and submitted to a multiplex polymerase chain reaction (PCR) directed to *Leishmania* kDNA (Passos et al. 1996) and to the IVS6 region of the *cacophony* gene in neotropical sandflies (Lins et al. 2002). The amplified products underwent dot blot hybridisation with a *L. (Viannia)* biotinylated probe (Pita-Pereira et al. 2005) (Suppl. data, Fig. 1). Negative (male insects) and positive (infected *Lutzomyia longipalpis* (Lutz & Neiva 1912) females fed on rabbit blood containing 2 × 10^5^ *L. braziliensis*/mL) controls were included. In two out of 173 samples, *Leishmania* kDNA product (120 bp) was visualised on stained agarose gel. Following hybridisation, the sensitivity increased and ten other samples revealed positive signals, beyond confirming the presence of *L*. (*Viannia*) DNA in both sandflies previously detected by gel electrophoresis (Suppl. data, Fig. 1). Thus, 12 out of 173 sandflies were positive, resulting in a detection rate of 6.94% for the finding of parasite DNA. The two positive samples first detected by PCR and agarose gel were related to forest area in the Bosque district (Figure - Area II), and corresponded to *Th. auraensis* (Mangabeira 1942) and *Pressatia* sp. (Mangabeira 1942). For the ten positive samples identified after hybridisation, two corresponded to *Evandromyia saulensis* (Floch & Abonnenc 1944) captured in the forest area of Chico Mendes Municipal Park (Figure - Area I). The remaining eight samples were identified as *Th. auraensis* from forest area of Bosque district (Area II). We did not find positive insects in the Moreno Maia settlement (Figure - Area III). All positive sandflies were collected in both periods of capture (April - rainy season, and October - dry season) with higher prevalence in October.

**Figure:**
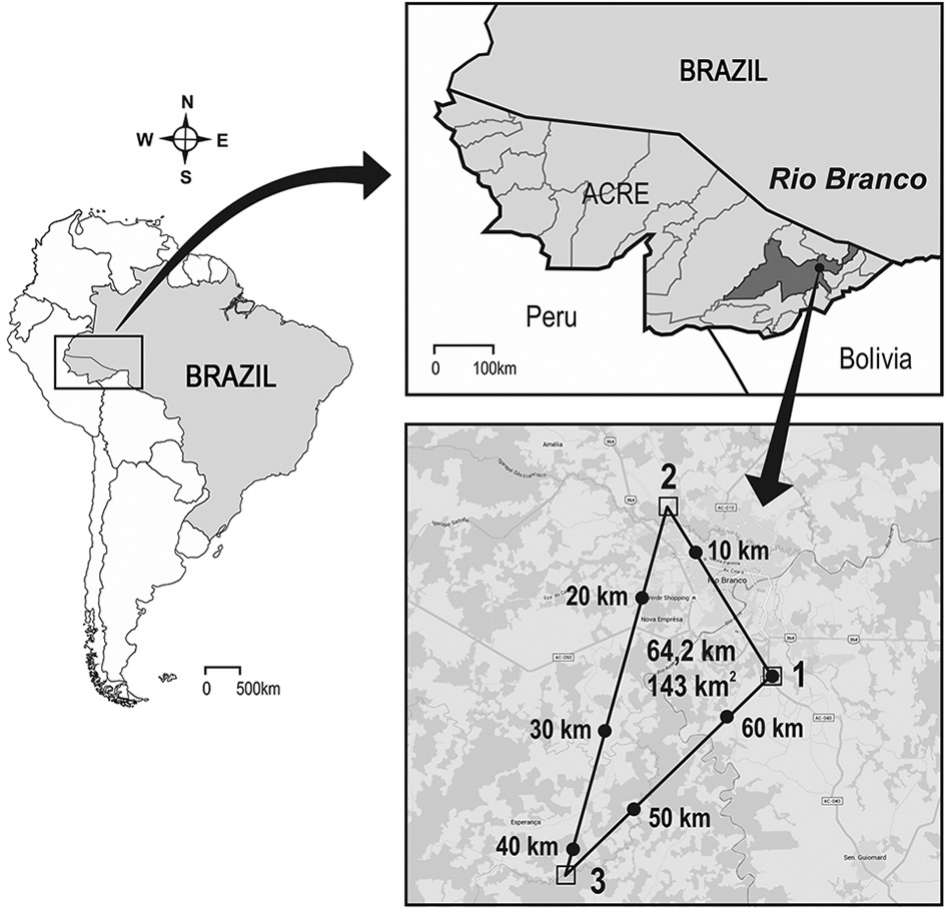
Municipality of Rio Branco, state of Acre, Brazil. Inset shows a satellite image with the distances among the three study areas where sandflies were collected (two collecting points were performed in each area; not indicated in the image). (1) Area I - Chico Mendes Municipal Park (10º02’135’’S, 67º47’716’’W) situated in the Vila Acre district. The park occupies an area of 52 ha and is located along the highway AC-040 and 10 km from the city of Rio Branco. It is considered one of the last remaining areas of primary forest with very important representative species of fauna and flora. (2) Area II - Bosque district (09º55’802’’S, 67º51’763’’W) represents an urban area located near the centre of Rio Branco, where houses have been constructed very close to the Amazon forest with the presence of domestic and wild animals in peri-domicile areas. (3) Area III - Moreno Maia settlement (10º10’357’’S, 67º55’505’’W) is situated along Transacreana Road and represents a rural area far away from the centre of Rio Branco, where the few existing residences are situated near forest with the maintenance of domestic animals in the peri-domicile areas. Source: Rio Branco (67º49’52”S and 9º59’11”W), Google Maps. Image from HM Nogueira Diniz; production service and image treatment, IOC, Fiocruz.

DNA samples from the 12 positive female sandflies were submitted to parasite species identification by amplifying the *hsp70 Leishmania* target, followed by cloning and sequencing the 234 bp fragments (da Graça et al. 2012). This specific region of the *hsp70* gene was confirmed to be a valid target for the diagnosis of ACL in humans with the capacity to distinguish among all species causing the disease in Brazil, after performing PCR-RFLP or sequencing (da Graça et al. 2012). At least three colonies for each positive insect sample were randomly picked up and sequenced. Consensus sequences were edited using the software package Phred/Phrap/Consed Version: 0.020425.c (University of Washington, Seattle, WA, USA) and those with Phred values above 20 were used as *contigs,* further assembled and aligned in the MEGA5 software (Tamura et al. 2007). Species identification was held through similarity analysis with the BLAST algorithm (Zang et al. 2000) by comparing with available sequences on GenBank. Since there is a remarkable number of accessible *hsp70* sequences for different *Leishmania* species and strains, such an approach allows the highest hit, i.e., E-value and identity, to reliably type a query’s species. Additionally, preliminary analysis of a large panel of *hsp70* (173 sequences) obtained from representative strains of different species of *Leishmania* circulating in Brazil that broadly covers the *L*. (*Viannia*) diversity (*Leishmania* Collection, Oswaldo Cruz Institute/CLIOC - unpublished data) was used as another source of comparison. For this, we performed clustering analysis to check similarity between samples.

The results of the 234 bp-*hsp70*C PCR and sequencing to identify among *L*. (*Viannia*) spp. found in the 12-sandfly DNA extracts, revealed five of them as *L*. (*V*.) *braziliensis* (Suppl. data, Fig. 2), based on highly stable polymorphisms presented among *L*. (*V*.) *braziliensis* DNA sequences, separating it from other species (da Silva et al. 2010, da Graça et al. 2012). These samples corresponded to *Pressatia* sp. (the only positive one) and *Th. auraensis* (three out of nine positive) collected in Area II, and *Ev*. *saulensis* (one out of two positive) from Area I. BLAST analysis revealed only one *Th. auraensis* specimen identical to *L*. (*V*.) *braziliensis* with 100% identity and E-value of 6.00E-75. The other four samples also matched with *L*. *braziliensis*, although with lower identity values varying between 95% and 99%, presenting E-values below 10 (the default threshold) (Suppl. data, Table). The DNA fragment used was short (234 bp) but informative to distinguish between *L*. (*Viannia*), regarding the current taxonomy for the subgenus, due to polymorphisms detected for each species within this fragment (da Silva et al. 2010, da Graça et al. 2012). In our study, the inconclusive results of sequencing the 234 bp-*hsp70*C fragment in seven out of 12 positive sandflies were due to the small DNA amount yielded by one single sandfly used for diagnosis and further parasite species identification, generating low-quality readings (Phred values below 20).

Although the number of trapped specimens was relatively low (456 sandflies in total; 256 females and 200 males), this Amazon Region presents a large variety of species, as observed in other areas of primary forest (Bejarano et al. 2002, Azevedo et al. 2008, Teles et al. 2013). *Th. auraensis* was the most frequent species collected (243/456 - 53.3%) and nine specimens were find harboring parasite kDNA out of the 96 non-fed blood *Th. auraensis* females analysed (9.4%). These results contrast with previous work performed in Acre, where this species comprised 19% of all captured sandflies (Azevedo et al. 2008). Curiously, the same species was also the most abundant (63%) in Madre de Dios, an Amazon Region in Peru bordering the state of Acre, but the prevalence of infection was lower (estimated to be 0.6% by kDNA-PCR performed in sandfly pools) (Valdivia et al. 2012). In the study area, authors identified *Th. auraensis* as a natural carrier of *L*. (*V*.) *lainsoni* and *L*. (*V*.) *braziliensis*, using a FRET-based real time PCR (Valdivia et al. 2012). Other investigation in Puno, Peru, showed that *Th. auraensis* could be anthropophilic (Arístides 1999), although this evidence requires confirmation (Fernandez et al. 1998). These findings highlight the need to infer the relevance of this species in the transmission of ACL in the Neotropics.

The other positive samples for *Leishmania* DNA found in the present study refer to *Ev. saulensis* (two positive out of nine submitted to molecular diagnosis - 22.2%) that constituted 3.5% of the total sandfly species collected, followed by *Pressatia* sp. (one positive out of four analysed - 25%) representing 3.3% of all species found in the study area. For the genus *Evandromyia* (Mangabeira 1941), there have been reports of *Ev. cortelezzii* (Brethes 1923) and *Ev. sallesi* (Galvão & Coutinho, 1940) naturally infected with *L.* (*L.*) *infantum* in Minas Gerais, Brazil (Carvalho et al. 2008, Saraiva et al. 2009). The complex *cortelezzii* has also been found infected by *L.* (*V*.) *braziliensis* in the municipality of Belo Horizonte, Minas Gerais (Saraiva et al. 2010). This finding was corroborated by an investigation in Chaco, Argentina in an active transmission area of ACL, reinforcing the hypothesis that members of the *cortelezzii* complex could act as vectors of *Leishmania* (Rosa et al. 2012). However, to date, there have been no reports of *Ev. saulensis* infected by *Leishmania* spp. This sandfly is widely distributed in South and Central America and has been frequently reported in Brazil, for instance in the Amazon Region (Young & Ducan 1994, Galati 1995). The identification of only one specimen of *Pressatia* sp. infected in our study corroborates recent report of *Leishmania* spp. infection in this same sandfly captured in a region of Peru bordering the state of Acre (Hugo Valdivia, personal communication). *Pressatia* sp., as other sandflies, are too similar to allow species discrimination based on morphological characteristics and may correspond to subpopulations in the early stages of speciation that have not yet evolved morphological differences (Pinto et al. 2015). There have been no reports of anthropophily or feeding habits for these two sandflies: *Ev. saulensis* and *Pressatia* sp.

In general, the finding of *L*. (*Viannia*) DNA in two *Ev*. *saulensis* with the confirmation of *L*. *braziliensis* in one specimen, as far as we know, corresponds to the first record of possible infection associated with this sandfly. Moreover, our study reveals for the first time, in Brazil, the identification of *Th*. *auraensis* and *Pressatia* sp. infected by *L*. (*Viannia*) parasites (where in three out of nine positive *Th*. *auraensis* and in the only positive *Pressatia* sp. the genetic material was confirmed as *L*. *braziliensis*), corroborating previous investigation carried out in Peru. The role of these species as vectors of parasites responsible for ACL remains to be established for better understanding the risk of New World tegumentary leishmaniasis transmission in the Neotropics.
