# Supplementary material for: First description of Leishmania (Viannia) infection in Evandromyia saulensis, Pressatia sp. and Trichophoromyia auraensis (Psychodidae: Phlebotominae) in a transmission area of cutaneous leishmaniasis in Acre state, Amazon Basin, Brazil
Source: Mem Inst Oswaldo Cruz. 2017 Jan;112(1):75–8. doi: 10.1590/0074-02760160283 (PMC5225531; doi:10.1590/0074-02760160283)
Supplement: Supplementary file 1 [file 0074-0276-mioc-112-1-0075-suppl01.pdf]

 Mem Inst Oswaldo Cruz, Rio de Janeiro, Vol. *112*(1): 1-2, January 2016

TABLE

Results for the major hits obtained after BLAST analysis of the 234 bp-hsp70C polymerase chain reaction and sequencing

Sample	Description	Max score	Total score	Query cover	E value	Max identity	Accession number
2 - *Evandromyia saulensis*	*Leishmania braziliensis* MHOM/BR/75/M2904 putative heat-shock protein hsp70 (LBRM_28_2970) mRNA, partial cds	261	261	78%	2,00E-66	96%	gi|389601962|XM_001566273.2
6 - *Trichophoromyia auraensis*	*L. braziliensis* MHOM/BR/75/M2904 putative heat-shock protein hsp70 (LBRM_28_2970) mRNA, partial cds	217	217	97%	2,00E-53	97%	gi|389601962|XM_001566273.2
3 - *Pressatia* sp	*L. braziliensis* MHOM/BR/75/M2904 putative heat-shock protein hsp70 (LBRM_28_2970) mRNA, partial cds	250	250	100%	3,00E-63	95%	gi|389601962|XM_001566273.2
11 - *Th. auraensis*	*L. braziliensis* MHOM/BR/75/M2904 putative heat-shock protein hsp70 (LBRM_28_2970) mRNA, partial cds	289	289	100%	6,00E-75	100%	gi|389601962|XM_001566273.2
5 - *Th. auraensis*	*L. braziliensis* strain MHOM/BR/2002/NMT-LTCP 14440-P clone B heat shock protein 70 (hsp70) gene, partial cds	379	379	100%	5,00E-102	99%	gi|316891027|GU368187.1

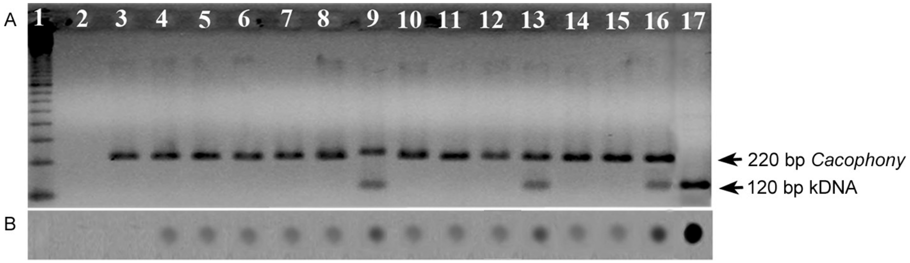

Fig. 1: natural infection by *Leishmania* spp in sandflies. (A) multiplex polymerase chain reaction (PCR) directed to the 220 bp fragment of the cacophony gene (sandflies) and the 120 bp of kDNA conserved minicircle sequence (*Leishmania* spp.) was performed individually in 173 sandfly DNA extracts. Amplified products were visualised on 2% agarose gel stained with ethidium bromide. 1: molecular weight 100 bp DNA ladder (Invitrogen); 2: negative control for PCR reaction (no DNA added); 3: negative control for DNA extraction (DNA extracted from male sandflies); 4-15: single female sandflies subjected to molecular diagnosis that revealed positivity in two samples (lines 9 and 13); 16: positive control (DNA extracted from laboratory-bred *Lutzomyia longipalpis* females); 17: positive control (DNA from cultivated *L. (V.) braziliensis*) [10 ng]. (B) dot blot hybridisation of PCR-amplified products with a specific byotinylated probe for *Leishmania* subgenus *Viannia*. 4-5: *Evandromyia saulensis* - Area of forest (area I); 6: *Pressatia* sp. - Area of forest (area II); 7-15: *Trichophoromyia auraensis* - Area of forest (area II); 16: positive control (DNA extracted from laboratory-bred *Lu. longipalpis* females); 17: positive control (DNA from cultivated *L. (V.) braziliensis*).

>11_*Trichophoromyia auraensis*
GATGCGTCGAAGTACGAGCAGGCCGACAAGATGCAGCGCGAGCGCGTGGAGGCGAAGAACGGCCTGGA
GAACTACGCGTACTCGATGAAGAACACGGTCTCCGACACGAACGTGTCCGGCAAGCTGGAGGA
>3_*Pressatia* sp
CAACCAGGAGGCGTCGAAGGAAGAGCAGGCCGACAAGATGCAGCGCGAGCGCGTGGAGGCGAAGAACG
GCCTGGAGAACTACGCGTACTCGATGAAGAACACGGTCTCCGACACGAACGTGTCCGGCAAGCTGGAC
GAGATCGA
>6_*Trichophoromyia auraensis*
CGTCGAAGGAAGAGCAGGCCGACAAGATGCAGCGCGAGCGCGTGGAGGCGAAGGACGGCCTGGAGAAC
TACGCGTACTCGATGAAGAACACGGTCTCCGACACGAACGTGTCCGGCAAGCTGGACGAGATCG
>5_*Trichophoromyia auraensis*
GATGCGTCGAAGTGCGAGCAGGCCGACAAGATGCAGCGCGAGCGCGTGGAGGCGAAGAACGGCCTGGA
GAACTACGCGTACTCGATGAAGAACACGGTCTCCGACACGAATGTGTCCGGCAAGCTGGAGGAGAGCG
ACAGGTCCGCGCTGAACTCGGCGATCGACACGGCGCTGGAGTGGCTGAAC
>2_*Evandromyia saulensis*
GATGCGTCGAAGGAAGAGCAGGCCGACAAGGTGCAGCGCGAGCGCGTGGAGGCGAAGAACGGCCTGGA
GAACTACGCGTACTCGATGAAGAACACGGTCTCCGACACGAACGTGTCCGGCAAGCTGKACGAGACG

Fig. 2: edited consensus sequences of the 234 bp-*hsp70*C polymerase chain reaction products.
